# Through the looking glass: self-reassuring meta-cognitive capacity and its relationship with the thematic content of voices

**DOI:** 10.3389/fnhum.2013.00213

**Published:** 2013-05-21

**Authors:** Charlotte Connor, Max Birchwood

**Affiliations:** Birmingham and Solihull Mental Health Foundation Trust, University of BirminghamBirmingham, UK

**Keywords:** voice-hearers, voice content, self-critical thoughts, self-reassuring meta-cognition, auditory hallucinations, voice power, voice expressed emotion

## Abstract

**Aims:** To examine the self-critical thoughts and self-reassuring meta-cognitive capacity of those who hear voices and explore whether they are associated with the theme of voice content and appraisals of voice power and voice expressed emotion.

**Method:** A cross-sectional design was used, combining semi-structured interviews and self-report measures. Data on symptomatology, self-critical thoughts and self-reassuring meta-cognitive capacity, thematic voice content, and appraisals of voice power and expressed emotion were collected from 74 voice-hearers in Birmingham, UK.

**Results:** Common themes of voice content reflected issues of shame, control, and affiliation. Controlling content was the most prevalent theme, however, no significant predictor of this theme was found; shaming thematic voice content linked with reduced capacity to self-reassure following self-critical thoughts. Voice-hearers with the greatest level of self-critical thoughts appraised their voices as powerful and high in voice expressed emotion.

**Conclusions:** Findings suggest that voice-hearers self-critical thoughts are reflected in the *type* of relationship they have with their voice. However, access to self-reassuring meta-cognitive capacity may serve as a protective factor for those who hear voices, resulting in more benign voice content. These findings highlight the importance of this specific meta-cognitive capacity and will inform future therapeutic interventions for the management of voices in this vulnerable group.

## Introduction

The “thoughts we have about our thoughts,” or our meta-cognitive capacity, helps us manage and control the flow of our cognition (Flavell, 1979). Inability to manage intrusive and unwanted cognitions, such as self-critical thoughts, intrinsic to the concept of shame, are commonly seen in those with depression (Gilbert and Miles, 2000; Gilbert et al., 2001; Connor and Birchwood, 2011a,b), who seem unable to access self-reassuring “thoughts about these thoughts” with which to alleviate the distress caused by them (Purdon and Clark, 2001).

Greater *frequency* of unwanted and intrusive thoughts and failure to cope with them has been associated with auditory hallucination proneness and are thought to underpin the phenomenon; a greater need to control their own “thoughts” associated with severity of hallucinations (Morrison and Baker, 2000; Varese and Bentall, 2011; Perona-Garcelan et al., 2012). The cognitive model of auditory hallucinations argues that voices arise due to misattribution of such unwanted intrusive thoughts to an *external* source in an unsuccessful attempt to dissipate the negative arousal that occurs on hearing them (Morrison et al., 1995). *Unwanted* and *intrusive* thoughts may be extremely distressing and voice-hearers may be particularly keen to dispel and attribute them to someone else, however, a recent study exploring the relationship between voice-hearers thoughts and voices has established that voice-hearers are able to distinguish voices from their everyday thoughts, reporting much less control over them in comparison to their own thoughts, the words heard seeming to reflect another “personality” (Hoffman et al., 2008).

The interpersonal nature of the voice/voice-hearer relationship is well documented, in about 40% of cases voices are described as “known” to the voice-hearer and voice-hearers in contact with mental health services commonly report hearing hostile and critical content which causes them much distress, including high levels of depression (Nayani and David, 1996; Leuder and Thomas, 2000). Such voice content can be likened to that heard in “real-world” high expressed emotion relationships which have been associated with increased rates of relapse in both psychosis and depression (van der Gaag et al., 2003), and linked with high levels of anxiety and low self-esteem (Bebbington and Kuipers, 1994; Barrowclough et al., 2003).

Parallels between general relationships and the voice/voice-hearer relationship have been observed by Birchwood et al., whose cognitive model of voices (Birchwood et al., 2000) revealed that voice-hearers appraisals of powerful and omnipotent voices were reflections of their own subordination and low status in general relationships; self-evaluative appraisals of low social rank identified as the primary predictors of depression in voice-hearers, independent of voice activity (Soppitt and Birchwood, 1997). Our recent work explored how these appraisals of voice power and expressed emotion might impact on the affective response to voices and revealed that those who appraised their voices as both powerful *and* high expressed emotion had the most severe depression and suicidal ideation (Connor and Birchwood, 2011a,b).

A disparity between voice content and the affective response to voices, however, has been observed, some voice-hearers, despite hearing powerful, critical and hostile voices, remaining emotionally unaffected by them, happy to maintain contact with their voices and feeling in control of the experience (Romme and Escher, 2000; Sanjuan et al., 2004; Jenner et al., 2008). This suggests that affective response to voices may be dependent on voice-hearers *mood* and *self-evaluation*.

Ability to self-reassure (vs. self-attack) intrusive and negative thoughts about ourselves and the capacity to disengage from the flow of thought is a distinct meta-cognitive capacity (Teasdale, 1999); this is the foundation for the therapeutic concept of mindfulness which has been shown to prevent relapse of depression (Gilbert, 2004). Access to such capacity may help to explain how some voice-hearers are happy to maintain contact with their voices, despite their hostile and critical nature; helping them dissipate the negative arousal that would otherwise be caused by being confronted by such negative content and enabling them to remain in control of the experience.

This paper focuses on the self-critical thoughts and self-reassuring capacity of voice-hearers in relation to thematic content of voices and their appraisals of their voice power and voice expressed emotion.

### Hypotheses

1. Voice-hearers' self-critical thoughts and self-reassuring meta-cognitive capacity will be associated with thematic content of voices.
2. Voice-hearers-hearers' self-critical thoughts and self-reassuring meta-cognitive capacity will be associated with appraisals of voice power and voice expressed emotion.

## Methods

### Design

A cross-sectional design was used. Data were collected from individual sessions with 74 participants, including structured and semi-structured interviews and self-report measures.

Individual examples of thematic voice content were examined using Framework Analysis, a deductive qualitative methodology designed for research which is applied or policy driven, highly appropriate for studies which have pre-set aims and objectives (Pope et al., 2000). Emergent categories of the themes were first indexed and then organized into thematic charts. All themes were rated independently by two qualitatively trained researchers. Individual ratings were then discussed and agreed upon to ensure concurrence.

### Inclusion/exclusion criteria

Eligibility criteria for participation in the study were a current diagnosis of schizophrenia or related disorder (WHO, 1992), in addition to the presence of auditory hallucinations for a minimum of the past 3 months. No exclusion criteria were used.

### Sampling

Participants were recruited from 15 Community Mental Health Teams based in urban National Health Service Trusts in Birmingham and Derby, UK. Interviews were conducted at a day care center, clinic, or in clients own home.

## Measures

### Symptomatology

#### Structured clinical interview for positive and negative syndrome scale

This widely used clinical measure assesses severity of positive and negative symptoms and general psychopathology (Opler and Lindenmayer, 1988). It is a 30-item interview rating severity of Positive symptoms (7 items; range, 7–49), Negative symptoms (7 items; range, 7–49), and General Psychopathology (16 items; range, 16–112). Regularly used in psychosis research, it takes approximately 30–45 min to complete and has good reliability and construct validity (Cronbach's alpha = 0.86). Reliability in the present sample = 0.68.

#### Calgary depression scale for schizophrenia

This structured interview is specifically designed to assess depression in schizophrenia *free of contamination by negative symptoms* (Addington et al., 1993). Consisting of eight scales reflecting the main symptoms of depression, each item is rated on a four-point rating scale; a higher overall score indicating a greater level of depression. Scores are classified into none, mild, moderate, or severe depression, using cut-off scores of 0–2 (no depression), 3–4 (mild depression), 5–6 (moderate depression), and 7+ (severe depression). It has excellent psychometric properties, and correlates highly with the Beck Depression Inventory (*r* = 0.87) (Beck et al., 1961), is easy to administer, discriminates well between those with and without major depression and has good internal consistency (coefficients of between 0.71 and 0.79). Reliability in the present sample = 0.75.

### Auditory hallucinations

#### Voice power differential scale (VPD)

This is a measure of the power differential between voice and voice hearer, and consists of 7 items rating participants perceived power, confidence, knowledge, strength, respect, ability to inflict harm, and superiority in relation to their voice (Birchwood et al., 2004). Using a 5-point Likert scale, it yields an overall total score; the greater the score, the greater the perceived power differential. Used frequently with voice hearers, it has good construct validity and reliability, a 1-week retest reliability of 0.8, and is internally reliable (Cronbach's alpha = 0.85). Reliability in the present sample = 0.82.

#### Level of expressed emotion scale (38 items, patient version) (LEE)

Although derived directly from EE theory (Hooley and Parker, 2006), this is not an alternative to the Camberwell Family Interview (the usual measure associated with rating *observed* EE), but functions as an easily administered, preferred self-report measure of *perceived* EE. It consists of 38 items and 4 scales: emotional support (19 items), intrusiveness (8 items), irritation (6 items), and criticism (5 items). It uses a 4-point Likert scale, yielding total scores for each constituent item and an overall score (range, 38–152), with a higher score reflecting a greater level of EE. The standardized cut-off for high EE was 80.45, and has high internal reliability (Cronbach's alpha = 0.91) (Cole and Kazarian, 1988). For the purposes of this study, the LEE was partially modified, in order to refer to voices rather than actual people, and instructed participants: “… the following statements describe the ways in which your voice may act toward you. Please indicate whether your voice has acted in these ways during the past 3 months.” All amendments were independently rated and agreed by both authors prior to implementation. Reliability in the present sample = 0.63.

### Self-critical thoughts and self-reassuring capacity

#### The other as shamer scale (OAS)—external shame

This measure was adapted from Internalized Shame Scale (Cook, 1993) and specifically measures “external shame” (Goss et al., 1994). It consists of 18 items rated on a 5-point scale according to the frequency of evaluations about how one thinks other people judge the self (0 = Never; 4 = Almost always), including statements such as: “I feel other people see me as not good enough,” “I think that other people look down on me” and “Other people put me down a lot.” It has high internal consistency (Cronbach's alpha = 0.92). Reliability in present sample = 0.77.

#### The function of self-criticizing scale (FSCS)—internal shame

Developed from clinical work focusing on self-critical thoughts and the ability to access self-reassuring meta-cognitions (Allan et al., 1994), this measure consists of 22 items rated on a 5-point scale according to how one feels the statements apply to the self (0 = Not at all like me; 4 = Extremely like me). It is made up of three factors: “inadequate self” (feeling internally put-down and inadequate following failure); “hated self” (a sense of self-dislike and aggressive/persecutory desire to hurt the self-following failure); and “reassured self” (a sense of encouragement and concern for the self when things go wrong) and includes statements such as: “I am easily disappointed with myself,” “I am able to remind myself about positive things about myself” and “I have a sense of disgust with myself.” It has high internal consistency (Cronbach's alpha = 0.86). Reliability in present sample = 0.69.

#### The forms of self-criticizing scale (FSCRS)—internal shame

This measure explores the *reasons* why people are self-critical. It consists of two factors: “self-correction” (wanting to improve performance and keep up standards), and, “self-persecution” (dislike and contempt for the self) (Allan et al., 1994). The measure begins with the statement “I get critical and angry with myself … ” and is followed by 21 possible reasons for self-attacking, including statements such as: “to make sure I keep up my standards,” “to stop myself being happy” and “to show I care about my mistakes,” It is rated on a 5-point scale (0 = not at all like me; 4 = extremely like me) and has good internal consistency (Cronbach's alpha = 0.92). Reliability in present sample = 0.67.

#### Analysis

Statistical Package for Social Sciences (SPSSW) for Windows, version 20 was used to analyse data. After testing to ensure data did not violate assumptions of normality, a correlational analysis was conducted to examine associations between symptom severity, self-critical thoughts, capacity to self-reassure, and appraisals of voice power and expressed emotion. This was followed by a series of logistic regression analyses analysis exploring the relative contribution of self-critical thoughts and self-reassuring meta-cognitive capacity in the prediction of voice power, voice expressed emotion, and thematic content of control, affiliation and shame. The latter were coded as “present” = 1 or “absent” = 0 for the purpose of the analysis.

## Results

One hundred and two clients were approached to participate in the study; 28 did not participate: 7 (6.8%) refused; 5(4.9%) were unable to participate due to relapse; 3 (2.9%) cancelled appointments; 3 (2.9%) denied hearing voices; 2 (1.9%) did not attend their appointments and were subsequently unable to be contacted. There was no feedback for 8 (7.8%) clients failed to respond to the initial request to participate. This resulted in a response rate of 73%, with 74 clients remaining in the final cohort.

Participants were predominantly male (59.5%), with a mean age of 43 years. 64.9% of them were white European, 20.3% Afro-Caribbean, 6.8% Indian-Asian, 4.1% mixed heritage, 2.7% British Pakistani, and 1.4% African. 55.4% of participants were single, and 86.5% were unemployed. 66.2% had been diagnozed with schizophrenia, 13.5% with depressive psychosis, 9.5% schizoaffective disorder, 2.7% affective disorder and 2.7% paranoid psychosis, 1.4% bi-polar disorder, 1.4% drug induced psychosis, 1.4% borderline personality disorder and 1.4% other. 71.6% had experienced their mental health problems for more than 10 years. Over half of the participants (58.1%) heard 5+ voices; for the purposes of this study only the dominant voice experienced was considered.

Symptomatology was assessed at entry into the study using the Structured Clinical Interview for Positive and Negative Syndrome Scale (SCI-PANSS). Mean scores revealed high levels of moderate hallucinatory behavior (Table 1). Forty-one (55.4%) participants were at least moderately depressed, with a mean CDSS score of 6.14 (*SD* = 4.64) (Table 2).

**Table 1:** **Mean scores for Positive symptoms (SCI-PANSS)**.

	**Mean score (SD)**
Positive symptoms (*n* = 74)^*^	**16.43 (4.30)**
Conceptual disorganization^**^	1.78 (0.99)
Hallucinations^**^	4.80 (0.77)
Excitement^**^	1.57 (0.77)
Grandiosity^**^	1.73 (1.15)
Suspiciousness/persecution^**^	2.66 (1.42)
Hostility^**^	1.08 (0.27)
Delusions^**^	2.76 (1.47)

* *Potential range of scores = 7–49*.

** *Potential range of scores = 1–7; 1 (absent), 2 (minimal), 3 (mild), 4 (moderate), 5 (moderate/severe), 6 (severe), 7 (extreme)*.

**Table 2:** **Depression, suicidal thinking, and hopelessness (Calgary depression scale)**.

**Depression score**	**Mean (SD) 6.14 (4.64)**
	**Number (%)**
**DEPRESSION^1^**	
No depression	15 (20.3)
Mild depression	18 (24.3)
Moderate depression	11 (14.9)
Severe depression	30 (40.5)
**SUICIDAL THOUGHT^2^**	
No suicidal thought	53 (71.6)
Suicidal thoughts	21 (28.4)
**HOPELESSNESS^3^**	
No hopelessness	40 (54.1)
Mild hopelessness	20 (27)
Moderate/severe hopelessness	14 (18.9)

1 *cut-scores: 0–2, none; 3–4, mild; 5–6, moderate; 7, severe*.

2 *cut-off scores: 0, none; 1–3, suicidal thoughts*.

3 *cut-off scores: 0, none; 1, mild; 2/3, severe*.

### Thematic content of voices

Seventy-three (99%) voice-hearers gave typical examples of what their voice said to them (see Appendix Table A1). Identification of thematic content of voices was based on the number of times each theme occurred, and corrected for overall output of themes. Eight overarching themes emerged all based on the spoken message reported by voice-hearers: controlling (45%), affiliative (38%), shaming (35%), threatening (22%), killing (16%), prosaic (12%), deceiving (5%), and obscene (3%). The three most frequent themes (controlling, affiliative, and shaming) appeared together on only one occasion, whereby a female voice-hearer heard the voices of her husband and father-in-law (affiliative) advising her on everyday things (controlling) and criticizing her for wanting a divorce (shaming). However, themes of control and shame often appeared alongside those which were affiliative; 28% of the time in both cases.

#### Hypothesis 1: voice-hearers' self-critical thoughts and self-reassuring meta-cognitive capacity will be associated with thematic content of voices

Correlational analysis found significant positive correlations between CDSS and OAS (external shame) (0.50; *p* = 0.01); FSCRS (self-persecution) (0.49; *p* = 0.01); FSCS (inadequate self) (0.60; *p* = 0.01) and (hated self) (0.54; *p* = 0.01). A negative correlation was also found with FSCS (self-reassuring capacity) (0.47; *p* = 0.01). This was followed by a regression analysis to examine the relative contribution of self-critical thinking and self-reassuring capacity in relation to thematic content of voices, controlling for CDSS, which was first force entered followed by OAS, FSCS, and FSCRS (Table 3). The best predictors of the thematic content of affiliation were OAS (external shame) (standardized Beta, −0.510; *p* = 0.001) and FSCS (self-persecution) (standardized Beta, −0.308; *p* = 0.028) (adjusted *R*-square = model 1: 0.090, significant *F* change = 0.005; model 2: 0.138, significant *F* change = 0.028). The only predictor of thematic content of shame was FSCRS (self-reassuring) (standardized Beta: −0.273; *p* = 0.019) (adjusted *R* square = 0.062). No significant predictor of the thematic content of control was found.

**Table 3:** **Results of logistic regression analysis examining voice power, expressed emotion, and thematic content of voices and their association with self-critical thinking and self-reassuring capacity**.

	**VPD**	**LEE**	**Controlling theme**	**Affiliative theme**	**Shaming theme**
CDSS	0.529	0.636	0.334	0.854	0.409
OAS	**0.047**	0.211	0.791	**0.001**	0.935
Self-correction	0.153	0.596	0.204	0.981	0.605
Self-persecution	0.099	0.420	0.544	**0.028**	0.408
Inadequate self	0.357	**0.008**	0.882	0.226	0.500
Hated self	**0.004**	0.342	0.433	0.249	0.976
Reassuring self	0.100	0.068	0.037	0.389	**0.019**

#### Hypothesis 2: voice-hearers hearers' self-critical thoughts and self-reassuring meta-cognitive capacity will be associated with appraisals of voice power and voice expressed emotion

Significant positive correlations were found between CDSS and VPD (0.35; *p* = 0.01) and LEE (0.37; *p* = 0.01). This was followed by a regression analysis to examine the relative contribution of self-critical thinking and self-reassuring meta-cognitive capacity in the appraisal of VPD and perceived LEE (Table 3). After force entering CDSS, followed by OAS, FSCS, and FSCRS this revealed that VPD was significantly predicted by OAS (standardized beta, 0.255; *p* = 0.047) and FSCRS “hated self” (standardized beta, 0.380; *p* = 0.004) (adjusted *R* square = model 1: 0.281, significant *F* change = 0.000; model 2: 0.312, significant *F* change = 0.047). The only predictor of LEE was FSCRS “inadequate self” (standardized Beta, 0.478; *p* = 0.008) (adjusted *R* square = 0.281).

## Discussion

This paper has examined the self-critical thoughts and self-reassuring meta-cognitive capacity of those who hear voices in order to understand their relationship with the thematic content of voices and appraisals of voice power and voice expressed emotion.

Our findings have revealed that, overall, the most common thematic content heard by our voice-hearers was controlling, whereby voices dominated the voice-hearer, often with orders, and instructions on how to live their life. This was, however, closely followed by shaming content, with voices commenting on the voice-hearers personal flaws and/or dishonorable behavior and affiliative content, whereby voices had some affiliative connection with the voice-hearer (either real-life or implied). These themes are indicative of voice omnipotence and the interpersonal nature of the experience, commonly reported by voice-hearer (Nayani and David, 1996; Leuder and Thomas, 2000; Gilbert et al., 2001; Connor and Birchwood, 2011a,b).

### The relationship between self-critical thinking, self-reassuring meta-cognitive capacity, and thematic content of voices

Whilst the majority of self-critical thoughts were not associated with thematic content of voices, affiliative thematic content (content regarding an interpersonal connection of some sort) was found to be heard by voice-hearers with the least self-critical thoughts, in particular, *external shame* (fear of negative evaluation by others) and *self-persecutory* thoughts. This suggests that voice-hearers who are not fearful of the negative evaluation of others in the real-world, and whose self-critical thinking doesn't serve a self- persecutory function, may be more likely to hear thematic content relating to interpersonal connection.

In addition, we revealed that the capacity to self-reassure, following self-critical thoughts, exerted an impact on the level of shaming content heard. We interpret these findings as preliminary evidence that voice-hearers self-critical thoughts and their capacity to self-reassure those thoughts determine the *theme* of what is heard.

### The relationship between self-critical thinking, self-reassuring meta-cognitive capacity, and appraisals of voice power and expressed emotion

Appraisals of voice power and high expressed emotion were most frequently reported by voice-hearers who expressed self-critical thoughts of self-hatred and inadequacy. Interestingly, no other self-critical thoughts were associated with these appraisals, suggesting that feelings of self-hatred and inadequacy may be particularly relevant in determining the *type* of interpersonal relationship voice-hearers have with their voice, particularly in relation to their social status and perceived level of emotional support; two dimensions of the voice/voice-hearer relationship found to be important predictors of depression and suicidal ideation in voice-hearers, in recent work (Connor and Birchwood, 2011a,b).

### Methodological issues

We acknowledge that the present findings are a merely a snapshot of a specific group of voice-hearers with high levels of depression and long-term mental health issues, therefore, any conclusions made will apply to this population only, who occupy the most distressed end of the voice hearing spectrum. Future research will be informed by a much wider cohort of voice-hearers, including those new to hearing voices, those of a younger age and include a non-clinical sample.

One of the main methodological issues with this study was the relatively small sample size (*n* = 74) which limited the power of the analyses and any sub-group comparisons.

Another issue was choice of cross-sectional design, which, whilst allowing us to collect the data swiftly and easily, may have been less effective than a longitudinal design through which we may have been able to establish whether our findings were supported over time.

Whilst our findings are encouraging, we were surprised at the absence of any association between *controlling* thematic content and self-critical thinking. One explanation for this finding could be that controlling thematic content (powerful, dominant, and often involving orders or instructions) is not a direct translation of self-critical thoughts of personal inadequacies and flaws but rather a reflection of voice-hearers subordination and low social rank in relation to others. In this context, and for future studies, it may be more useful to differentiate between several dimensions of self-critical thinking.

We must not, however, dismiss the possibility that our failure to find a greater number of associations between thematic content and self-critical thinking is evidence of no *direct* relationship between the two. Future research should consider whether voice-hearers self-critical thinking might serve a more indirect purpose, not in *determining* thematic content or appraisals of power and EE, but serving to *maintain* the type of relationship they have with their voices. However, our earlier work exploring abuse and dysfunctional relationships in childhood found that voice-hearers who had experienced emotional abuse in childhood had the greatest levels of present-day shame cognitions (Connor and Birchwood, 2011a,b). Unsupportive early relationships are well-known to predispose individuals to later interpersonal difficulties (Collins and Read, 1990; Bretherton and Munholland, 2008). Such dysfunctional relationships in childhood determine how we regard ourselves and, how we believe others will respond to us. Self-reassuring meta-cognitive skills are theorized to originate through positive and reaffirming interpersonal early attachments with caregivers (Bowlby, 1982) who become increasingly recognized as reliable sources of affiliative support, equipping individuals with the skills to successfully negotiate future interpersonal relationships. These findings suggest that self-critical thoughts and self-reassuring capacity may be present *prior to* the development of auditory hallucinations and whilst they may serve a role in maintaining the relationship with voices, may also be implicated in their inception.

A recent review of the meta-cognitive beliefs account of auditory hallucinations by Varese and Bentall (2011), concluded that there was insufficient robust evidence to suggest a direct association between meta-cognitive beliefs and auditory hallucinations, and argue that meta-cognitive beliefs may have more in common with co-morbid symptoms; for example, thought insertion or delusions (Morrison, 2001; Linney and Peters, 2007). We should treat our findings, therefore, as a proof-of-principle study in order to provide preliminary testing of the hypotheses which will need to be confirmed in further work. Our initial findings are, however, promising and our application of strict and careful procedures to ensure concordance and inter-rater reliability we hope will help us build on these initial foundations.

### Therapeutic implications

Auditory hallucinations have been suggested to arise due to misattribution of unwanted and intrusive thoughts, emerging in an attempt to negate their impact (Morrison et al., 1995); the meta-cognitive beliefs voice-hearers hold about their intrusive thoughts suggested to be associated with the *distress* caused by the experience (van der Gaag et al., 2003; Brett et al., 2009; Hill et al., 2012). The present findings support and add to this literature, revealing that in a cohort of voice-hearers with long-term mental health issues, self-critical thoughts and *incapacity* to self-reassure has an impact on the *type* of interpersonal relationship they have with their voice.

“Compassionate Mind Training” (CMT) is aimed at reducing self-critical thoughts and has been extremely successful in *increasing* feelings of self-reassurance and acceptance in non-psychotic patients with depression, resulting in *significant reductions* in shame and self-critical thoughts (Gilbert and Procter, 2006). We hope this study will inform the design of similar therapeutic interventions for depression and suicidal ideation in this vulnerable group.

### Conflict of interest statement

Professor Birchwood and Dr.Connor are part funded by the National Institute of Health Research CLAHRC (Collaboration for Leadership in Applied Health Research and Care) Birmingham and The Black Country. The views contained herein are those only of the authors.

## Appendix

**Table A1:** **Examples of voice themes**.

**Examples**				
Husband and father-in-law advising her on everyday things; criticizing her for wanting a divorce	Shame	Control	Affiliation	Bullying
Woman she used to work with asking how she is	Affiliation			
Very distressed woman asking for their help	Affiliation			
Male and female strangers telling her to kill her family	Control			Bullying
Male and female strangers swearing and saying, he's by the car and I'm going to kill you	Threat			Bullying
Brother and sister-in-law telling he to come to new Delhi on holiday	Affiliation			
Ex-boyfriend and nephew asking her where she's going and discussing her appearance	Shame	Control	Affiliation	Bullying
People calling out her name and cats crying	Affiliation	Prosaic		
Friends and family saying you are going to hell, people don't love you, people don't like you	Shame		Affiliation	Bullying
Woman he knew saying come and save me and look for me; handful of male and female strangers pretending to be woman and saying I'm alright now, it's okay, trying to make out that they are her and to prevent him from rescuing her	Affiliation	Deception	Control	

Definitions of thematic content:

*Affiliation – content regarding an interpersonal association, relationship or connection with someone known or suggestion of desired affiliation*.

*Shame – content regarding dishonor or ignominy; this could include appearance, sexuality, or other behavior*.

*Control – content regarding power or domination; this could include orders and instructions*.

*Killing – content regarding killing of self or others*.

*Deception – content regarding voice pretending to be someone else*.

*Threat – content regarding violent and/or threatening behavior (not killing)*.

*Prosaic – mundane content*.

*Obscene – content of a sexual nature*.
